# Robust Pedestrian Detection by Combining Visible and Thermal Infrared Cameras

**DOI:** 10.3390/s150510580

**Published:** 2015-05-05

**Authors:** Ji Hoon Lee, Jong-Suk Choi, Eun Som Jeon, Yeong Gon Kim, Toan Thanh Le, Kwang Yong Shin, Hyeon Chang Lee, Kang Ryoung Park

**Affiliations:** Division of Electronics and Electrical Engineering, Dongguk University, 26 Pil-dong 3-ga, Jung-gu, Seoul 100-715, Korea; E-Mails: easygns@dgu.edu (J.H.L.); jjongssuk@dgu.edu (J.-S.C.); jeunsom@dgu.edu (E.S.J.); csokyg@dongguk.edu (Y.G.K.); lethanhtoan@dgu.edu (T.T.L.); skyandla@dgu.edu (K.Y.S.); leehc@dongguk.edu (H.C.L.)

**Keywords:** pedestrian detection, visible light image, thermal image, dual camera system

## Abstract

With the development of intelligent surveillance systems, the need for accurate detection of pedestrians by cameras has increased. However, most of the previous studies use a single camera system, either a visible light or thermal camera, and their performances are affected by various factors such as shadow, illumination change, occlusion, and higher background temperatures. To overcome these problems, we propose a new method of detecting pedestrians using a dual camera system that combines visible light and thermal cameras, which are robust in various outdoor environments such as mornings, afternoons, night and rainy days. Our research is novel, compared to previous works, in the following four ways: First, we implement the dual camera system where the axes of visible light and thermal cameras are parallel in the horizontal direction. We obtain a geometric transform matrix that represents the relationship between these two camera axes. Second, two background images for visible light and thermal cameras are adaptively updated based on the pixel difference between an input thermal and pre-stored thermal background images. Third, by background subtraction of thermal image considering the temperature characteristics of background and size filtering with morphological operation, the candidates from whole image (CWI) in the thermal image is obtained. The positions of CWI (obtained by background subtraction and the procedures of shadow removal, morphological operation, size filtering, and filtering of the ratio of height to width) in the visible light image are projected on those in the thermal image by using the geometric transform matrix, and the searching regions for pedestrians are defined in the thermal image. Fourth, within these searching regions, the candidates from the searching image region (CSI) of pedestrians in the thermal image are detected. The final areas of pedestrians are located by combining the detected positions of the CWI and CSI of the thermal image based on OR operation. Experimental results showed that the average precision and recall of detecting pedestrians are 98.13% and 88.98%, respectively.

## 1. Introduction

In recent years, with the development of intelligent surveillance systems, the need for the accurate detection of pedestrians using cameras has increased. Intelligent surveillance systems should detect pedestrians at all times, and this is required to guarantee good detection performance in a variety of environments. However, accurate detection is a very difficult problem because of the variability of pedestrian’s appearance and various outdoor environments. Despite this situation, most previous research only used a single camera system of visible light or thermal camera, and their performance is not sufficient.

Previous research can be categorized as single camera-based research and dual camera-based research. The former uses the method of detecting pedestrians using a visible light camera [1,2,3,4,5], or thermal infrared camera [6,7,8,9,10,11,12,13,14,15,16,17,18,19,20,21,22,23,24,25].

In the research based on visible light cameras, the information from temporal differencing was used to detect pedestrians [1]. Other studies used wavelet templates [2], adaptive boosting (AdaBoost) detectors [3,4], and histograms of oriented gradient (HOG) with support vector machines (SVM) [5] for the detection of people. However, they have the limitation that their accuracies in detecting people are affected by various factors such as non-uniform illumination, shadow, and low external light during the evening and night. To overcome this problem, the research based on thermal camera is considered as an alternative.

In previous studies on pedestrian detection, the HOG method [8,9,14,15,16,18], classification based on SVM [10], AdaBoost method [6], soft-label boosting algorithm [7], contour saliency map (CSM) [12,19], CSM template matching [20], shape and appearance-based detection [22,23], spatiotemporal texture vectors [21], and boosting framework [24] was used. In addition, background information for detecting people based on a Gaussian background-subtraction approach [12,19,20], texture change [21], expectation minimization (EM) [22,23], and image averaging [24] was used. In other research [25], a particle filter framework and histogram based on the intensity-distance projection space for pedestrian detection was adopted. These methods, based on thermal cameras, are less affected by illumination change, shadow, and low external light during the evening and night. However, their performances are affected by high background temperatures in the daytime, which makes it difficult to discriminate people from the background. 

To overcome these problems, research has been done using dual camera systems. Bertozzi *et al.*, proposed a method based on stereo thermal cameras [17], but their method did not solve the problem of high background temperatures in the daytime. Zhao *et al.*, proposed the method of tracking people by combining visible and thermal cameras [26]. However, they had experiments with images where the people were close to the camera only indoors (where the visible light and thermal image do not include the effects by non-uniform illumination, shadow, and low external light in the outdoors during the evening and night). In addition, they did not show the quantitative accuracies of people detection. In [27], they used both visible and thermal cameras. However, their experiments were done only at night (where the thermal image does not include the effects by high background temperatures in daytime) nor did they show the quantitative accuracies of people detection. St-Laurent *et al.*, also proposed the method of combining visible and thermal cameras [28]. They used the co-axial structure of visible and thermal cameras, where the axes of the two cameras are identical. The additional glass beamsplitter with indium-tin-oxide (ITO) coating was used for the co-axial structure, which can reflect thermal energy while transmitting visible waves. However, the camera viewing angle of thermal and visible cameras is usually large in order to be used for outdoors surveillance systems, which inevitably makes the size of the glass beamsplitter large and consequently the size of the system also increases. 

To overcome the problems of these previous works, we propose a new method of pedestrian detection using a dual camera system by combining visible light and thermal cameras, which are robust to various outdoor environments such as mornings, afternoons, nights and rainy days. We implement the dual camera system where the axes of visible light and thermal cameras are parallel in the horizontal direction, from which the images captured by the two cameras are aligned based on the geometric transform matrix. Two background images for visible light and thermal cameras are adaptively updated when the pixel difference between an input thermal image and a pre-stored thermal background image is smaller than threshold. By background subtraction of thermal image considering the temperature characteristics of background and size filtering with morphological operation, the candidates from whole image (CWI) in the thermal image is obtained. The positions of CWI (obtained by background subtraction and the procedures of shadow removal, morphological operation, size filtering, and filtering of the ratio of height to width) in the visible light image are projected on those in the thermal image by using the geometric transform matrix, and the searching regions for pedestrians are defined in the thermal image. Within these searching regions, the candidates from the searching image region (CSI) of pedestrians in the thermal image are detected. The final areas of pedestrians are located by combining the detected positions of the CWI and CSI of the thermal image based on OR operation. 

Table 1 compares the previous and proposed methods for pedestrian detection.

The remainder of this paper is structured as follows: In Section 2, we describe the proposed system and method. Then, the experimental environment and results are shown in Section 3. Finally, we present the conclusions in Section 4.

**Table 1:** Comparison of previous and proposed methods.

Category		Method	Advantages	Disadvantage
Single camera-based method	Using visible camera [1,2,3,4,5]	By using spatial or temporal information only in visible light image	The performance of people detection in daytime of high temperature is higher due to the high resolution and quality of visible light image	The performance is affected by non-uniform illumination, shadow, and low external light during evening and night
	Using thermal camera [6,7,8,9,10,11,12,13,14,15,16,18,19,20,21,22,23,24,25]	By using spatial or temporal information only in thermal image	The performance is less affected by illumination change, shadow, and low external light during evening and night	The performance is affected by high background temperatures in daytime
Dual camera-based method	Using stereo thermal cameras [17]	By using spatial information in stereo thermal images	Higher performance of people detection than the single camera-based method	The performance is affected by high background temperatures in daytime
	Using co-axial structure of visible and thermal cameras [28]	Elaborately co-aligned structure of visible light and thermal cameras is used		Additional device of beamsplitter is required, and large beamsplitter increases the system size
	Using visible light and thermal cameras	People detection inconstrained environment such as indoor [26] or night-time [27]		Not showing quantitative accuracies of people detection in various environments such as high background temperatures in daytime, non-uniform illumination, and shadow in outdoors
		People detection in unconstrained environments (proposed method)	Robust to various environments without the additional device for combining two cameras	Lower processing speed than single camera-based method due to processing of two camera images

## 2. Proposed Method

### 2.1. Hardware Device for Pedestrian Detection and Camera Calibration

Surveillance systems often employ either near-infrared (NIR) or far-infrared (FIR) cameras. FIR cameras capture the image signal based on thermal radiation that is represented in the wavelength of 8–12 μm [28]. Therefore, it is called long wavelength infrared light (LWIR). NIR cameras capture the image signal based on light whose wavelength is much shorter (0.75–1.4 μm) than LWIR. FIR cameras acquire images without additional illuminator whereas NIR cameras usually require additional NIR illuminators to capture images, especially in night. Therefore, the NIR camera capturing distance is limited due to the limitation of the illumination distance, and large illuminator is required in order to capture the image at a distance. In addition, according to the Z distance of the object to be captured, the illumination angle should be adjusted by the illuminator’s lens so as not to saturate the object by the illuminator. In addition, the impact of absorption and scattering of fog is known to be less severe in the LWIR light than NIR one [28], which is one of important factor to be considered when the surveillance system is used in outdoors. Therefore, our dual camera system employs an FIR camera instead of an NIR one. 

**Figure 1:**
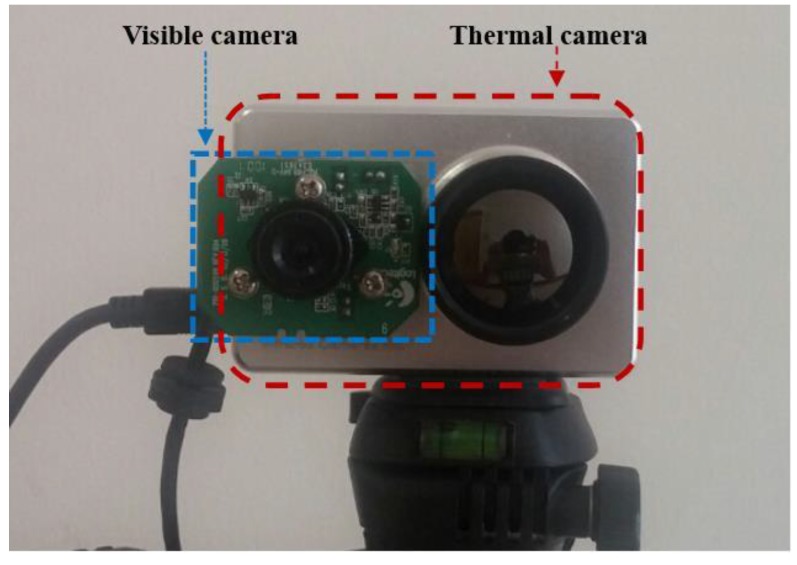
Proposed dual camera system.

Figure 1 shows the dual camera system used in our research. We create the dual camera system by combining visible light and thermal cameras. A commercial thermal camera of ICI 7320 is used [29]. It can capture an image of 320 × 240 pixels having a resolution of 14 bits in the wavelength range of 7–14 μm with the measurement accuracy of temperature of ±1°. A 25 mm lens is used in the thermal camera, and the field of view (FOV) of the thermal camera is 18° and 14° in the horizontal and vertical directions, respectively. The dimension (height × width × depth) and weight of the thermal camera are 2.1" × 3.2" × 0.5" and approximately 148 g, respectively. 

In order to reduce the size and cost of our system, a small, low-cost conventional web-camera is used as the visible light camera [30]. The FOV of the visible light camera is 20.4° and 15.4° in the horizontal and vertical directions, respectively. Due to the limitation of data transfer by universal serial bus (USB) 2.0 and capturing two images at the same time, our system acquires the visible light image of 640 × 480 pixels and the thermal image of 320 × 240 pixel at the capturing speed of 30 frames per sec. In order to reduce the image disparity between the two cameras, we make the two axes of visible light and thermal cameras parallel in the horizontal direction with minimum horizontal distance between the two cameras as shown in Figure 1. 

**Figure 2:**
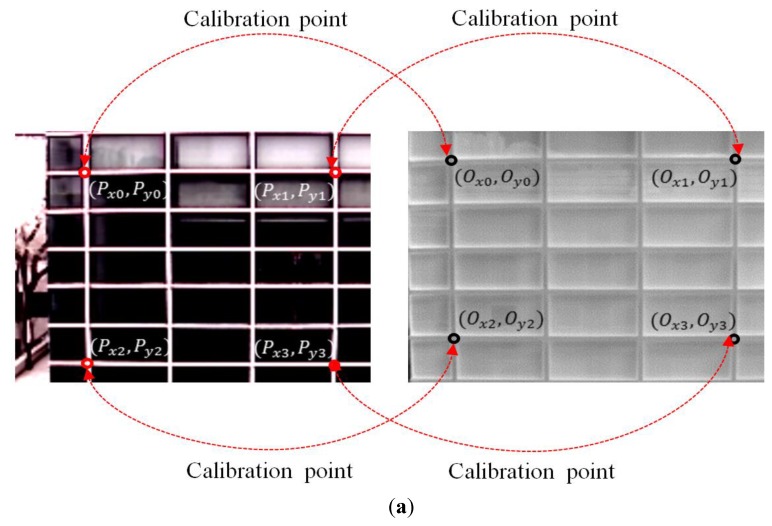

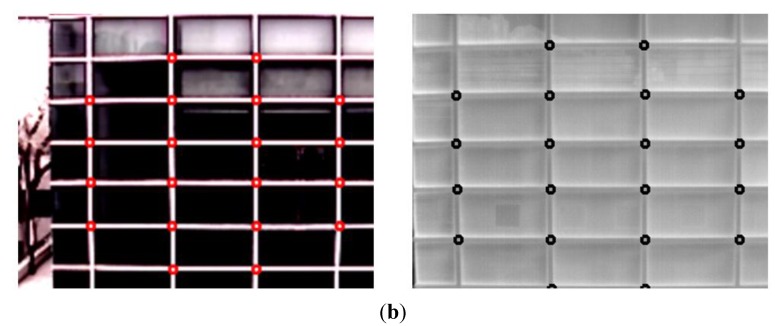
Calibration between two cameras based on geometric transform and accuracy measurements of the calibration. (**a**) Examples of calibration points used for calculating the matrix of geometric transform in the visible light (left) and thermal (right) images, respectively; (**b**) Points used for calculating the calibration error in the visible light (left) and thermal (right) images, respectively.

Then, the coordinates of two images by visible light and thermal cameras are set to be coincident by camera calibration based on geometric transform as shown in Equation (1) and Figure 2a. As shown in Equation (1), a pair of four points are required for obtaining the eight unknown parameters (*a*, *b*, … *h*) in the matrix of geometric transform, and this pair of four ground-truth points are used in the images by visible light and thermal cameras as shown in Figure 2a. These ground-truth points are manually obtained in our research because the procedure of obtaining the matrix of geometric transform is performed once, when the two cameras are combined, and it is not necessary to repeat this procedure irrespective of the subsequent setup locations of our dual camera system.

(1) $$\left[\begin{array}{c} \begin{array}{cc} O_{x0} & O_{x1}\\ O_{y0} & O_{y1} \end{array}\;\begin{array}{cc} O_{x2} & O_{x3}\\ O_{y2} & O_{y3} \end{array}\\ \begin{array}{cc} 0\; & 0\\ 0\; & 0 \end{array}\;\begin{array}{cc} 0 & \;0\\ 0 & \;0 \end{array} \end{array}\right]=\left[\begin{array}{c} \begin{array}{cc} a & b\\ e & f \end{array}\;\begin{array}{cc} c & d\\ g & h \end{array}\\ \begin{array}{cc} 0 & 0\\ 0 & 0 \end{array}\;\begin{array}{cc} 0 & 0\\ 0 & 0 \end{array} \end{array}\right]\left[\begin{array}{c} \begin{array}{cc} P_{x0}\; & P_{x1}\\ P_{y0}\; & P_{y1} \end{array}\;\begin{array}{cc} P_{x2}\; & P_{x3}\\ P_{y2}\; & P_{y3} \end{array}\\ \begin{array}{cc} P_{x0}P_{y0} & P_{x1}P_{y1}\\ 1 & 1 \end{array}\;\begin{array}{cc} P_{x2}P_{y2} & P_{x3}P_{y3}\\ 1 & 1 \end{array} \end{array}\right]$$

(2) $$\left[\begin{array}{c} \begin{array}{c} O{\text{′}}_{x}\\ O{\text{′}}_{y} \end{array}\\ \begin{array}{c} 0\\ 0 \end{array} \end{array}\right]=\left[\begin{array}{c} \begin{array}{cc} a & b\\ e & f \end{array}\;\begin{array}{cc} c & d\\ g & h \end{array}\\ \begin{array}{cc} 0 & 0\\ 0 & 0 \end{array}\;\begin{array}{cc} 0 & 0\\ 0 & 0 \end{array} \end{array}\right]\left[\begin{array}{c} \begin{array}{c} P{\text{′}}_{x}\\ P{\text{′}}_{y} \end{array}\\ \begin{array}{c} P{\text{′}}_{x}P{\text{′}}_{y}\\ 1 \end{array} \end{array}\right]$$

In order to measure the calibration error, a pair of 20 ground-truth points (which are not used for obtaining the matrix of geometric transform) is used as shown in Figure 2b. These ground-truth points are also manually obtained in our research. Based on the matrix of geometric transform (from visible light to thermal images) of Equation (2), we obtain the positions of the 20 points (of the visible light image) which are projected into those (the positions of 20 points) in the thermal image. Then, the average root mean square (RMS) error is calculated as the calibration error with the projected positions and 20 ground-truth points in the thermal image. In addition, based on the inverse matrix of geometric transform (from thermal to visible light images), we obtain the positions of the 20 points (of the thermal image) which are projected into those (the positions of 20 points) in the visible light image. Similarly, the average RMS error is calculated as the calibration error with the projected positions and 20 ground-truth points in the visible light image. Detail explanations and results of measuring calibration errors are shown in Section 3.1.

### 2.2. Proposed Method for Detecting Pedestrian

**Figure 3:**
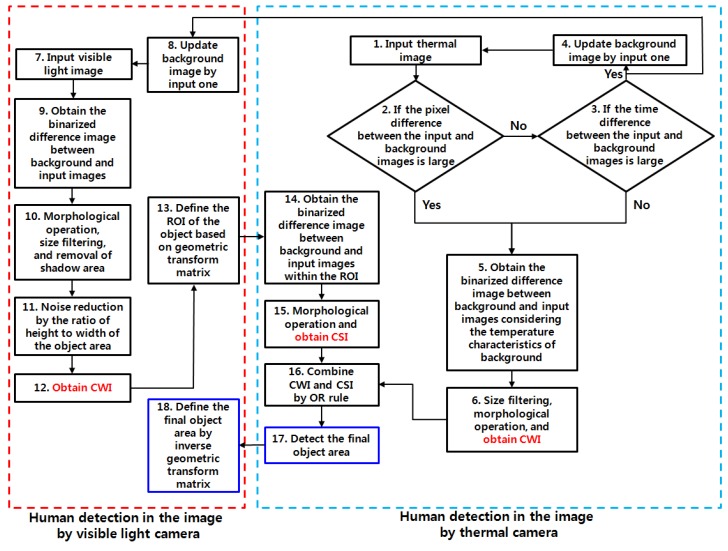
Flow chart of the proposed system.

Figure 3 shows the detecting procedures used in our research. As shown in Figure 3, our method is composed of two parts of human detections in the images by visible light and thermal cameras, respectively. In our system, two images, using thermal and visible light cameras, are acquired at the same time (Steps (1) and (7)). Then, the pixel difference between the background (which is already stored at the initial setup of the system) and input images is calculated. If the pixel difference value is less than threshold and the time difference (between the background and current input images) is large (as shown in Steps (2) and (3)), the background image is updated by the current input image (Step (4)). If not, the pixel difference image is binarized by adaptive threshold based on the temperature characteristics of background image (Step (5)). Through the size filtering and morphological operation, the CWI is obtained (Step (6)). 

In case the background thermal image is updated by the current input image (Step (4)), the background (visible light) image is also updated by the current input image (Step (8)). Then, the binarized difference image between the background and input thermal images is obtained as shown in the Step (9). With this image, the human object area is detected through morphological operation, size filtering, removal of shadow area, and noise reduction as shown in Steps (10)–(12). With the detected area, the corresponding region of interest (ROI) of the object region in the thermal image is defined based on geometric transform matrix (Step (13)), and this matrix is obtained in advance by camera calibration as explained in Section 2.1. Then, the binarized difference image between the background and input thermal images is obtained within this ROI as shown in the Step (14). With this image, the CSI is obtained by morphological operation (Step (15)), and the final area of human in the thermal image is obtained by combining the CWI (which is obtained in Step (6)) and CSI based on OR rule (Steps (16) and (17)). The object region in the visible light image is also obtained by an inverse geometric transform matrix based on the final area of the human in the thermal image as shown in Step (18).

### 2.3. Proposed Method for Detecting Pedestrian

As explained in Section 2.2 and Figure 3 (Steps (2)–(4), and (8)), two background images using visible light and thermal cameras are adaptively updated if the pixel difference value between the input and background thermal images is less than threshold, as shown in Equation (3), and there is a large time difference between the background and current thermal input images.

(3) $$\text{Background update}=\;\{\begin{array}{ll} true,\; & if\;\sum_{x}\sum_{y}|Frame_{x,y}\left(t\right)-\;Frame_{x,y}\left(t-n\right)|>Th\\ false,\; & otherwise\; \end{array}$$

where $Frame_{x,y}(t)$ is the pixel value (at the position (*x*, *y*)) of current thermal image at time *t*, $Frame_{x,y}(t-n)$ is the pixel value (at the position (*x*, *y*)) of background thermal image at time *t − n*. Our system determines whether the background image is updated by the input image based on only the thermal image as shown in Steps (2) and (3) of Figure 3. This is because using both the images by visible light and thermal cameras takes a great deal of processing time. In addition, it is usually more difficult to determine whether background image is updated by the input when using the visible light image because the visible light image is more affected by various factors of shadow by sunlight, illumination change, low illumination at evening or night, *etc*., compared to the thermal light image.

When obtaining the binarized difference image between background and input images (Step (5) of Figure 3), the temperature characteristics of background is considered in our method as follows. At first, we empirically determined *th*1 and *th*2 (of Equation (4)) which represent the maximum and minimum temperature of pedestrian, respectively. If the pixel intensity (P(x, y)) of background at the position ((x,y))  belongs to the range from *th*2 and *th*1 as shown in Equation (4), we can assume that the pixel difference between the pedestrian and background is small because the pixel intensities of background and pedestrian belong to the same range from *th*2 and *th*1. Therefore, we define this pixel position as *label2* and use smaller threshold for binarizing the difference image between background and input images in this case as shown in Equation (4). Other cases mean that the pixel difference between the pedestrian and background is large. Therefore, the pixel position is defined as different labels of *label1* and *label3*, and larger threshold for binarization is used based on the temperature characteristics of background as shown in Equation (4). Using the background subtraction based on this adaptive thresholding for binarization, we can obtain the candidate region of pedestrian, which is less affected by the temperature of background.

(4) $$\{\begin{array}{ll} \left(x,\;y\right)=\;label1, & if\left(P\left(x,y\right)\geq th1\right)\;\\ \left(x,y\right)=\;label2, & else\;if(th1>P(x,y)>th2)\\ \left(x,y\right)=\;label3, & else\;if\left(P\left(x,y\right)\leq th2\right)\; \end{array}$$

where (x,y) is x and y positions of input and background images, and P(x, y) is the pixel intensity (at the position (*x*, *y*)) in a background image. The *th*1, *th*2, and *th*3 are the thresholds for classifying the pixel intensity of P(x, y) (temperature of background).

This scheme of Equation (4) is used when obtaining the binarized image by the background subtraction with the input and pre-stored background images. Assuming that the input and background images are I(x, y) and P(x, y) of 2 × 2 pixels, respectively. If the P(0,0)≥th1, th1>P(0,1)>th2, P(1,0)≤th2, and P(1,1)≤th2, the positions of (0,0), (0,1), (1,0), and (1,1) have the *label*1, *label*2, *label*3, and *label*3, respectively. Then, we use the different thresholds for binarization according to the *label*1, *label*2, and *label*3 as shown in Equations (5)–(7).

(5) $$\{\begin{array}{l} B\left(\text{x},\text{y}\right)=1\;if\left(\left|I\left(x,\;y\right)-P\left(x,y\right)\right|\geq thr1\right)and\;if\left(\left(x,y\right)==label1\right)\\ B\left(\text{x},\text{y}\right)=0\;if\left(\left|I\left(x,\;y\right)-P\left(x,y\right)\right|<thr1\right)and\;if\left(\left(x,y\right)==label1\right) \end{array}$$

(6) $$\{\begin{array}{l} B\left(\text{x},\text{y}\right)=1\;if\left(\left|I\left(x,\;y\right)-P\left(x,y\right)\right|\geq thr2\right)and\;if\left(\left(x,y\right)==label2\right)\\ B\left(\text{x},\text{y}\right)=0\;if\left(\left|I\left(x,\;y\right)-P\left(x,y\right)\right|<thr2\right)and\;if\left(\left(x,y\right)==label2\right) \end{array}$$

(7) $$\{\begin{array}{l} B\left(\text{x},\text{y}\right)=1\;if\left(\left|I\left(x,\;y\right)-P\left(x,y\right)\right|\geq thr3\right)and\;if\left(\left(x,y\right)==label3\right)\\ B\left(\text{x},\text{y}\right)=0\;if\left(\left|I\left(x,\;y\right)-P\left(x,y\right)\right|<thr3\right)and\;if\left(\left(x,y\right)==label3\right) \end{array}$$

where *thr*1, *thr*2, and *thr*3 are the thresholds for binarization. 1 and 0 represent the white and black pixel, respectively. That is, *B*(0,0) is determined as 1 or 0 based on the Equation (5) because the position of (0,0) has *label*1. In addition, *B*(1,1) is determined as 1 or 0 based on the Equation (7) because the position of (1,1) has *label*3. By conclusion, the procedure of Equation (4) is performed before image binarization. Then, the image binarization is done according to the *label*1, *label*2 and *label*3 as shown in Equations (5)–(7).

In order not to lose the thermal information of the image, the binarized pixel difference image is obtained using the original thermal image of 16 bits. Because the thermal image usually includes salt and pepper noises, the binarized pixel difference image is obtained after the median filtering of the image.

### 2.4. Obtaining CWI and CSI from the Thermal and Visible Light Images

**Figure 4:**
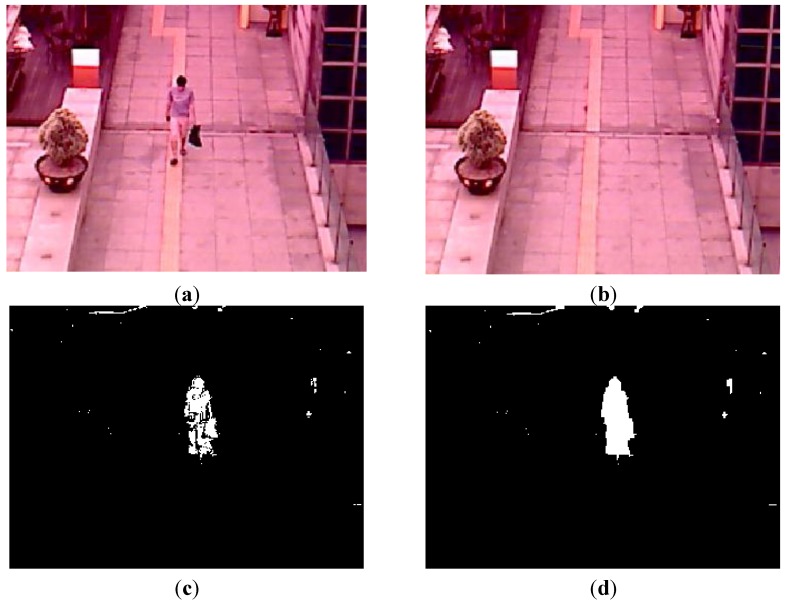

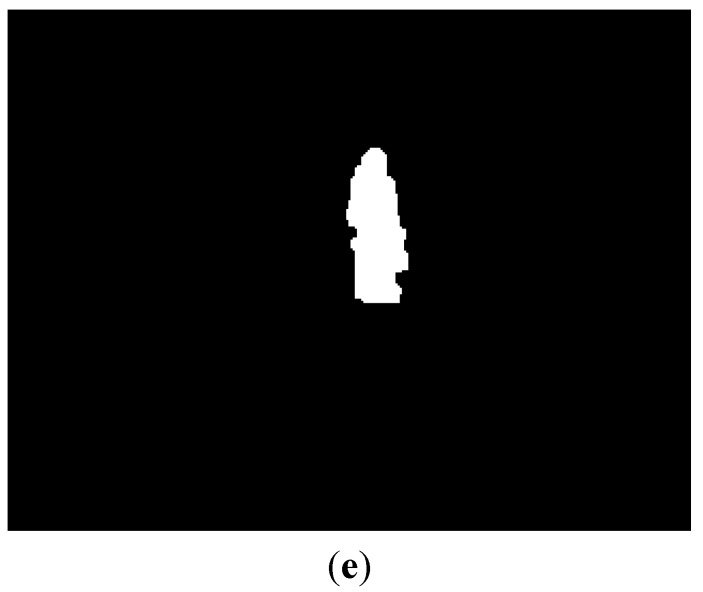
Examples of results by morphological operation and size filtering with the binarized difference image between background and input visible light images. (**a**) Input visible light image; (**b**) Background image; (**c**) The binarized difference image between background and input visible light images; (**d**) Result image by morphological operation; (**e**) Result image by size filtering.

**Figure 5:**
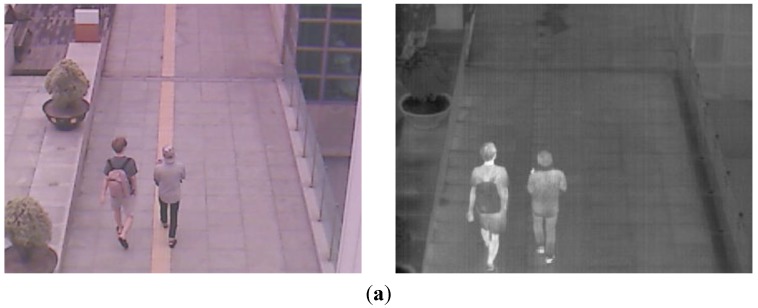

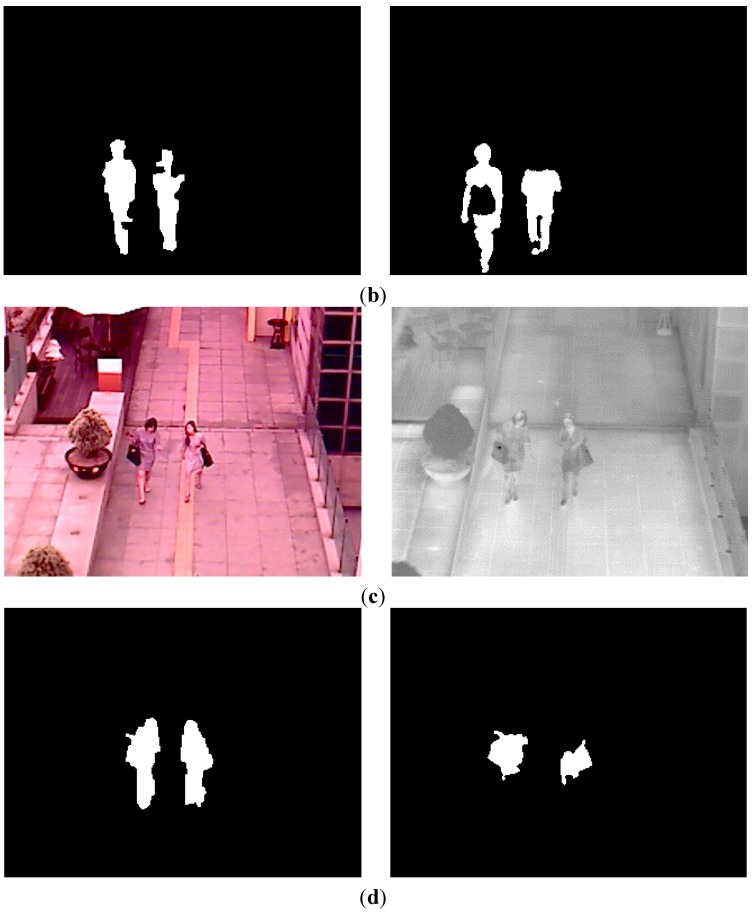
Examples obtained from CWI. (**a**) First example of the current input images by visible light (left figure) and thermal cameras (right figure) in morning; (**b**) Detected CWIs in visible light (left figure) and the thermal input image (right figure) of (a); (**c**) Second example of the current input images by visible light (left figure) and thermal cameras (right figure) at afternoon; (**d**) Detected CWIs in visible light (left figure) and the thermal input image (right figure) of (c).

With the binarized pixel difference image, the accurate CWI is located through component labeling, size filtering, and morphological operations. The CWI of the visible light image is obtained through background subtraction, morphological operation, size filtering, removal of shadow area, and noise reduction (based on the ratio of height to width of the detected object region) as shown in Steps (9)–(11) of Figure 3. Detail explanations of morphological operation and size filtering are as follows. With the binarized difference image between background and input visible light images (Figure 4c), morphological operation of erosion and dilation [31] is performed two times, and the result image is obtained as shown in Figure 4d. Through the morphological operation, holes inside the human candidate region can be filled as shown in Figure 4d. Then, we perform the size filtering that removes the candidate region (whose size is smaller than the threshold) as noise. As shown in Figure 4e, the noises except for human candidate region are removed by size filtering. The right images of Figure 5b,d show the examples of the detected CWI in the thermal image. Because the CWI of thermal image is combined with the CSI based on OR rule as shown in Step (16) of Figure 3, our system uses strict threshold with which the CWI is detected without additional incorrectly detected regions.

In most cases, the shadow region is difficult to be discriminated from the human area. To remove the shadow, we use the hue, saturation, and intensity information of the current and background images. We compared the same candidate region with the detected human in both the input and background images. If the hue and saturation values of one pixel within this candidate region of the input image are similar to those of the corresponding pixel of background image, and the intensity values of same pixel in input and background images are different, this pixel is determined as shadow region. This is based on the principle that the color information of shadow region in the input image is similar to that of corresponding background area whereas the intensity information in the input image is different (lower) from that of corresponding background area [32]. The left images of Figure 5b,d show the examples of the detected CWI in the visible light image. Because the CWI of visible light image is only used to define the ROI of the object in the thermal image as shown in Step (13) of Figure 3, our system uses the rough threshold with which the CWI is detected even though additional incorrectly detected regions occur.

**Figure 6:**
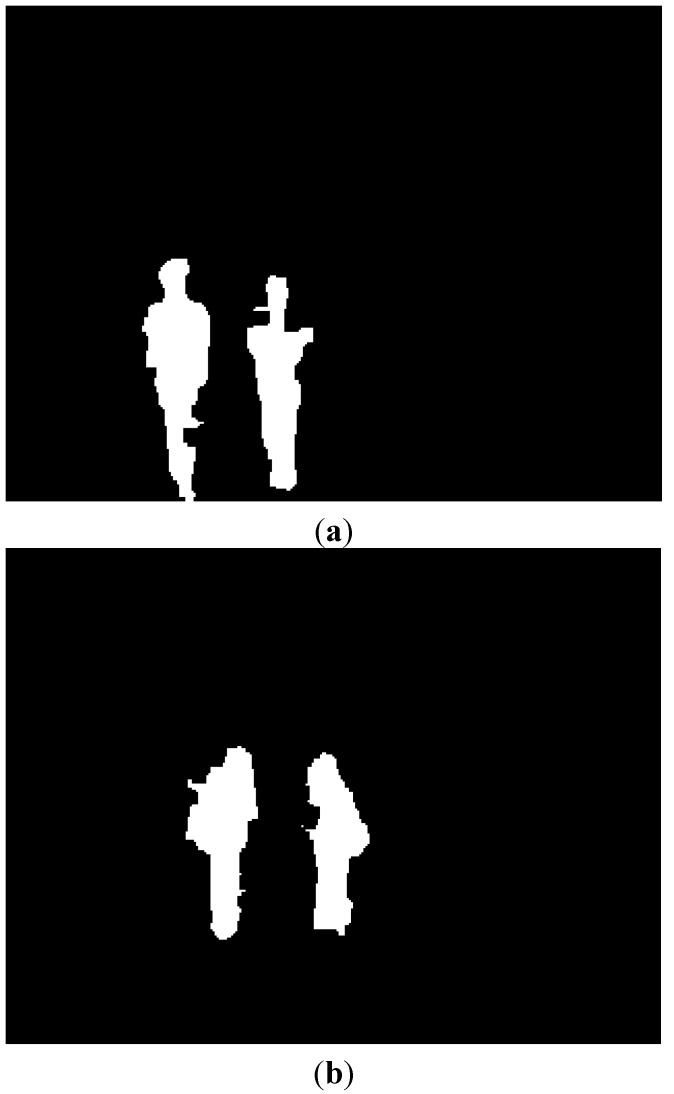
Examples of the obtained CSI in the thermal image. (**a**) The CSI obtained from both Figure 5a and the left image of Figure 5b; (**b**) The CSI obtained from both Figure 5c and the left image of Figure 5d.

Then, the ROI of the object in the thermal image from the CWI in the visible light image is defined as shown in Step (13) of Figure 3. As explained in Section 2.1, the two axes of visible light and thermal cameras are parallel in the horizontal direction with minimum horizontal distance between the two cameras in our system. In addition, we obtain the geometric transform matrix by camera calibration as shown in Figure 2a, and the corresponding position of the ROI of the visible light image can be obtained in the thermal image with the matrix. With the four corner positions of the ROI, the corresponding positions in the thermal image are calculated using the matrix and Equation (2). Within the ROI, which is defined the corresponding positions in the thermal image, the CSI of object region is detected by background subtraction and morphological operations as shown in Figure 6a,b.

**Figure 7:**
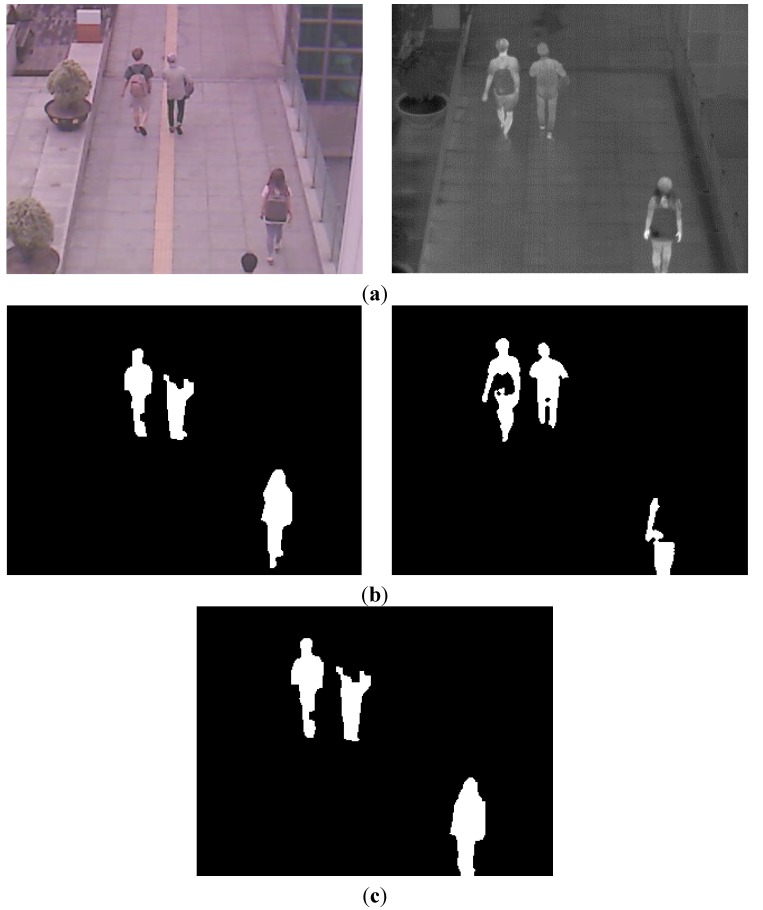
Examples of CWI and CSI. (**a**) Visible light and thermal images in the morning; (**b**) Results of CWI in visible light (left figure) and thermal (right figure) images; (**c**) Result of CSI in a thermal image.

However, using only the CSI (of Figure 6a,b) or the CWI (the right images of Figure 5b,d) can degrade the accuracy of human detection as shown in Figure 7. If the color of the human’s clothes are similar to that of background, as shown in the left image of Figure 7a, the object detection based on background subtraction in our system is not correct. Finally, this causes the errors in the visible light image of CWI (the left image of Figure 7b) and corresponding CSI (which is obtained based on the CWI in the visible light image) (Figure 7c). To overcome this problem, our system combines the CWI obtained in the thermal image (right image of Figure 7b) and the CSI (Figure 7c) (Step (16) of Figure 3). Detail explanations are included in Section 2.5.

### 2.5. Detecting the Final Human Area by Combining the CWI and CSI

**Figure 8:**
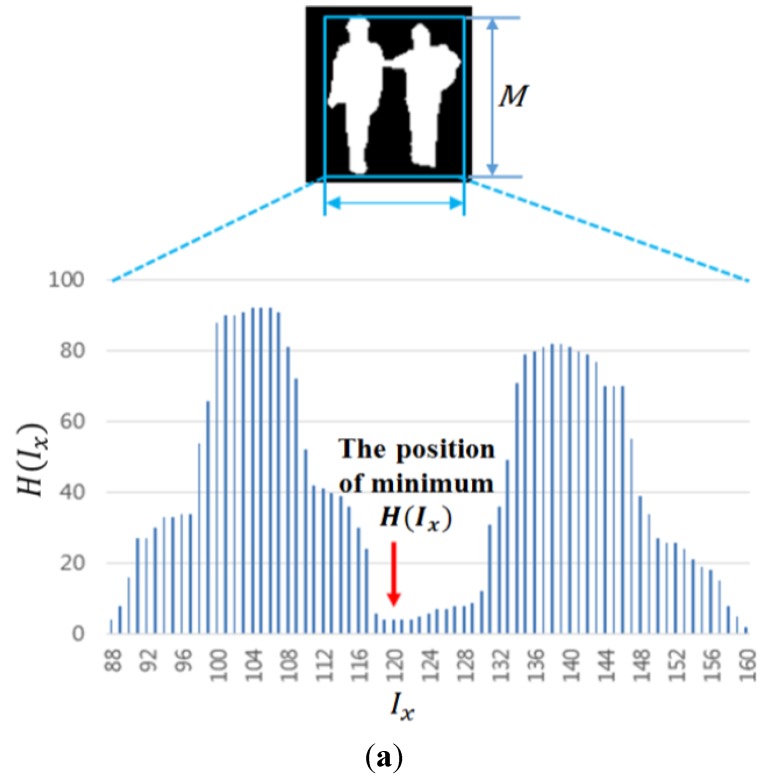

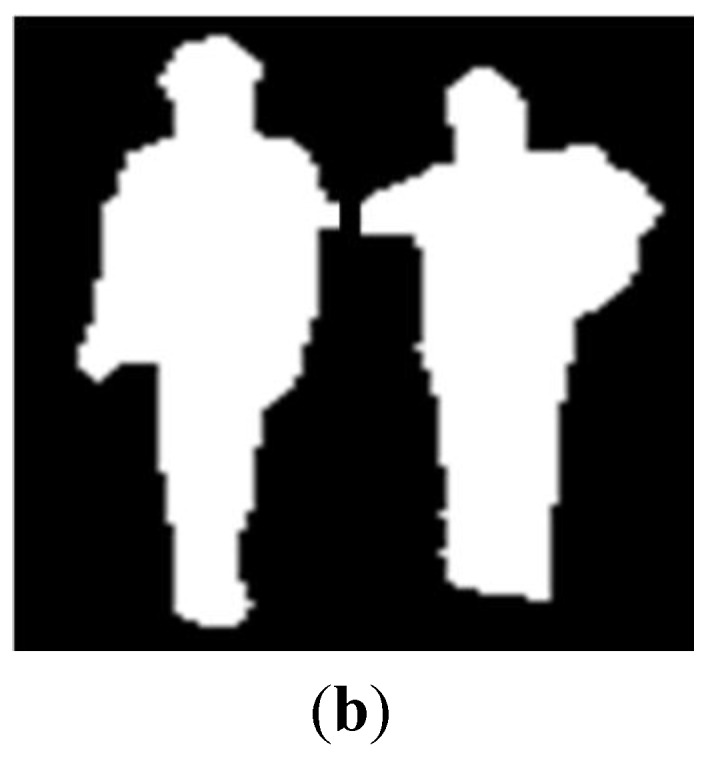
Separation of one candidate region into two areas based on the horizontal histogram. (**a**) Detected candidate region and its horizontal histogram; (**b**) The separation result of one candidate region into two areas.

As explained in Section 2.4, our system combines the CWI obtained in the thermal image and the CSI based on OR rule of Equation (8) in order to more accurately detect humans.

(8) $${\text{Result image}}_{\text{b}}=\;{\text{CWI}}_{\text{b}}\;||{\text{CSI}}_{\text{b}}$$

where the subscript b denotes the binarized image. With the result image, the final human area is detected after the morphological operation and histogram projection. Detail explanations about histogram projection are as follows. Horizontal histograms of each candidate region are obtained to determine whether one candidate region should be divided into two areas as shown in Figure 8. In detail, if the size of a detected region is greater than a threshold or the ratio of the height to width is not satisfied with the condition, the candidate region is divided into two parts based on the horizontal histogram information. The horizontal histogram is obtained by Equation (9):

(9) $$H(I_{x})=\overset{M-1}{\underset{y=0}{\sum }}B(P(x,y))$$

where *P*(*x*, *y*) is the binarized pixel value (white or black) at a location (*x*, *y*) within the candidate region (the combined area of CWI and CSI by OR rule in the thermal image). *B*(·) becomes one if *P*(*x*, *y*) is white, otherwise zero. *M* is the height of the candidate region. *I_x_* is the horizontal index of the candidate region within the image as shown in Figure 8a. As indicated in Figure 8a, if the minimum value of *H*(*I_x_*) is lower than the threshold, one candidate region is divided into two parts at the position (*I_x_*), as shown in Figure 8b. Like this procedure, vertical histogram projection is also performed with the combined areas of CWI and CSI if the size of a detected region is greater than a threshold or the ratio of the height to width is not satisfied with the condition. If the minimum value of vertical histogram is lower than the threshold, one candidate region is divided into two parts at the position of the minimum value in the vertical direction.

**Figure 9:**
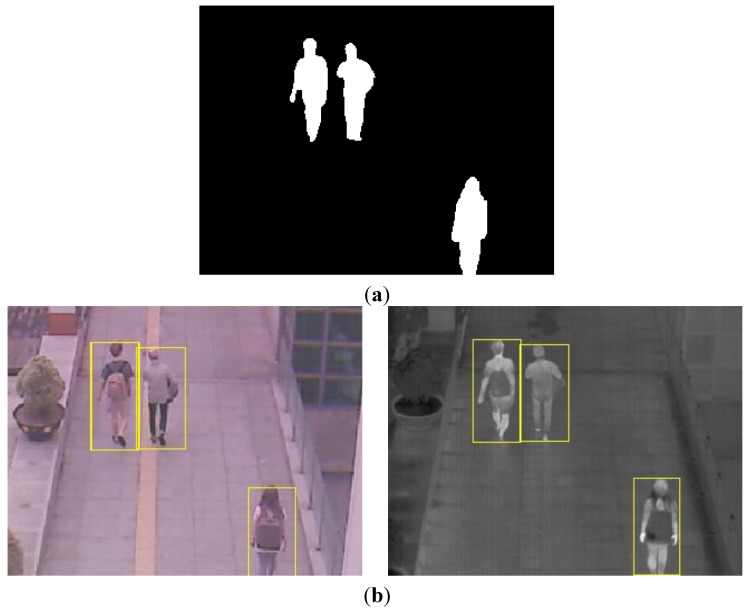
Examples of combined image of CWI and CSI, and the final result of human detection. (**a**) Combined image of CWI (right image of Figure 7b) and CSI (Figure 7c); (**b**) Final result of human detection.

The final results of human detection are shown in Figure 9. The area of human in the visible light image is also defined by the inverse geometric transform matrix as shown in Step (18) of Figure 3. 

## 3. Experimental Results

### 3.1. Experiment Environment and Calibration Error

Although there exist an open database for human detection of thermal images [33] or those for human detection of visible light images [34], there is no open database (for human detection) which is obtained by both visible light and thermal cameras. Therefore, we used the database that was collected by our dual camera system. The data acquisition for the experiments was performed using a laptop computer and the dual cameras (Figure 1). All the images using visible light and thermal cameras were acquired simultaneously. The laptop computer was equipped with a 2.50 GHz CPU (Intel (R) Core (TM) i5-2520M) and 4 GB RAM. The proposed algorithm was implemented using a C++ program using Microsoft foundation class (MFC) and OpenCV library (Version 2.3.1). To obtain the image, we installed the system of our dual cameras at the position whose height was 20 m from the ground outdoors. The total number of images used in the experiment (database I) was 2000. The sizes of the visible light and thermal images are 640 × 480 pixels and 320 × 240 pixels, respectively. These images were obtained in various environments such as mornings (22.6 °C), afternoons (26.0 °C), nights (21.1 °C), and rainy days (19.1 °C and precipitation of 48.0 mm). We captured the images where people naturally move without any instruction from us. Therefore, there exist various cases that some people are close together, cluttered, or separated, *etc*. in our database.

**Figure 10:**
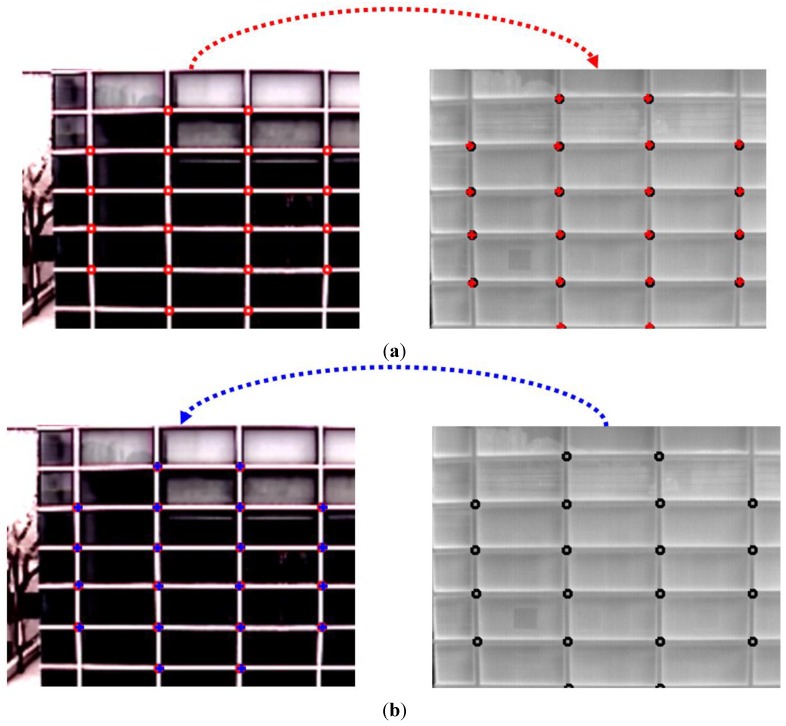
Calibration error between the two cameras (example 1). Left and right figures of (**a**,**b**) are visible light and thermal images, respectively. In each image, the circle and crosshair represent the ground-truth and calculated points, respectively (**a**) When using the geometric transform matrix (from visible light to thermal images); (**b**) When using the geometric transform inverse matrix (from thermal to visible light images).

As the first experiment, we measured the calibration error between the visible light and thermal cameras based on the geometric transform as explained in Section 2.1 and Figure 2. As explained in Section 2.1, a pair of 20 ground-truth points (which are not used for obtaining the matrix of geometric transform) is used as shown in Figure 2b. These ground-truth points are manually obtained in our research. Based on the matrix of geometric transform (from visible light to thermal images), we obtain the positions of 20 points (of the visible light image) which are projected into those in the thermal image. Then, the average RMS error is calculated as the calibration error with the projected positions and 20 ones in the thermal image. In addition, based on the inverse matrix of geometric transform (from thermal to visible light images), we obtain the positions of 20 points (of the thermal image) which are projected into those in the visible light image. Then, the average RMS error is calculated as the calibration error with the projected positions and 20 ones in the visible light image. The results are shown in Figure 10 and Table 2. As shown in Figure 10 and Table 2, the calibration error is less than 1.2 pixels, and we find that our calibration between the two cameras is accurate.

**Figure 11:**
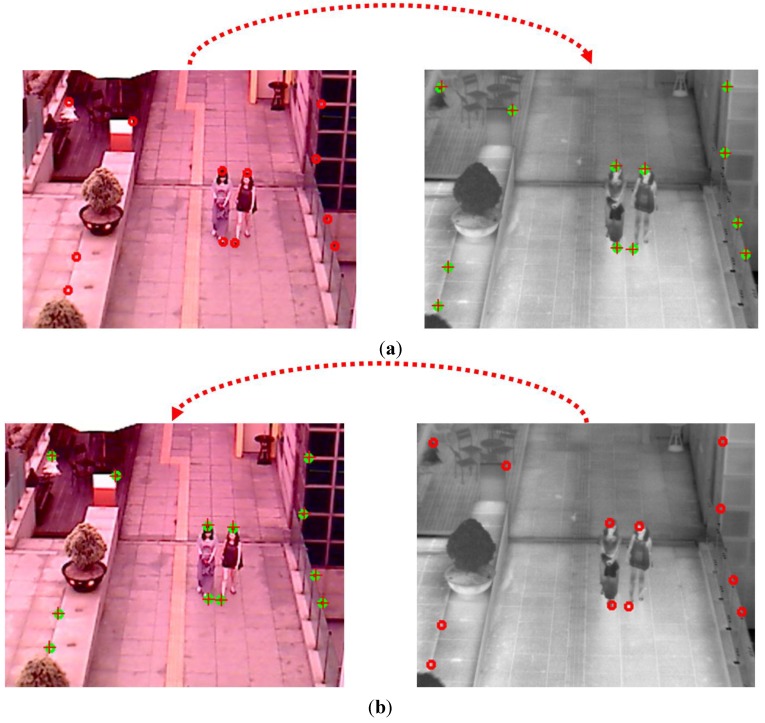
Calibration error between the two cameras (example 2). Left and right figures of (**a**,**b**) are visible light and thermal images, respectively. In each image, the circle and crosshair represent the ground-truth and calculated points, respectively (**a**) When using the geometric transform matrix (from visible light to thermal images); (**b**) When using the geometric transform inverse matrix (from thermal to visible light images).

In addition, we measure the calibration error with the points on real objects (the tiptoe and head top points of two persons as shown in Figure 11) and those on a different plane than the pavement (the other points except for the tiptoe and head top points of two persons as shown in Figure 11). As shown in Figure 11 and Table 3, the average RMS error with the points on real objects and those on a different plane than the pavement is similar to that with the points on the calibration object of Figure 10 and Table 2.

**Table 2:** Result of calibration errors of Figure 10 (unit: pixel).

Applying Geometric Transform Matrix		Average Pixel Error		Average RMS Error
From	To	X Direction	Y Direction	
Visible light image	Thermal image	1	0.5	1.12
Thermal image	Visible light image	1.15	0.25	1.18

**Table 3:** Result of calibration errors of Figure 11 (unit: pixel).

Applying Geometric Transform Matrix		Average Pixel Error		Average RMS Error
From	To	X Direction	Y Direction	
Visible light image	Thermal image	0.88	0.67	1.11
Thermal image	Visible light image	1.09	0.45	1.18

### 3.2. Detection Result of Human Area

**Figure 12:**
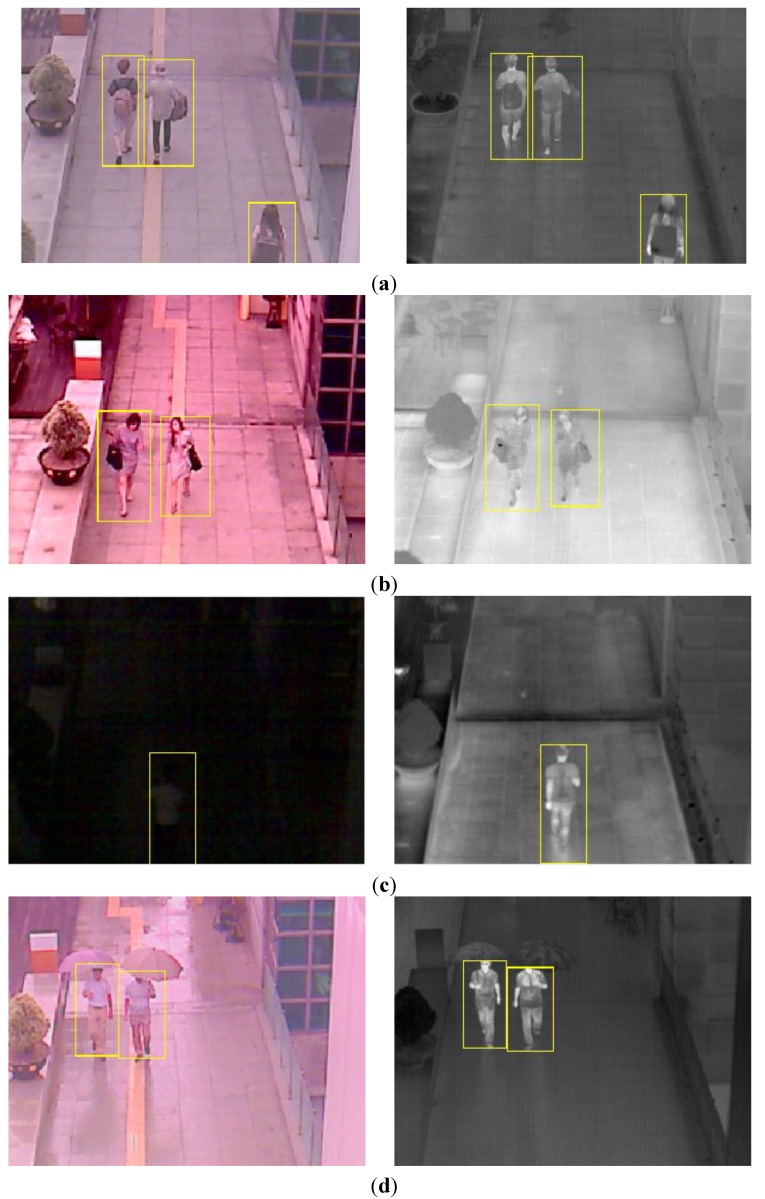
Example of detection results in various environments. (**a**) Detection result in the morning; (**b**) Detection result in the afternoon; (**c**) Detection result at night; (**d**) Detection result on a rainy day.

As the next experiment, we measured the accuracies of human detection. Some examples of human detection are shown in Figure 12. In each Figure 12a–d, the detected boxes of the left figure (visible light image) are shown by the inverse geometric transform matrix and the detected results of thermal image as shown in the Step (18) of Figure 3. As shown in Figure 12, we can find that our system detects humans in various environments. Even in the case when the human is not seen in the image by the visible light camera at night as shown in the left image of Figure 12c, our system can detect the human area successfully.

As the next experiment, we quantitatively measured the accuracy of human detection. For this, the bounding boxes of the human areas were manually depicted in the images as ground truth regions. The detection results were evaluated based on Pascal Criteria [13,35], which determine the true or false positives by calculating the overlap of the bounding box and a ground truth box as shown in Equation (10).

(10) $$Overlap_{d,g}=\;\frac{area(B_{d}\cap B_{g})}{area(B_{d}\cap B_{g})}$$

where $B_{d}$ denotes the box detected by our system. $B_{g}$ is the ground truth box. $\left(B_{d}\cap B_{g}\right)$ denotes the intersection of $B_{d}$ and $B_{d}$. $\left(B_{d}\cap B_{g}\right)$ is their union [35].

Based on Equation (9), we obtain the true positive (TP) and false positive (FP) of the detection. The TP is the case that the human region is correctly located as a human. The FP is the case that the background region is incorrectly located as a human. We quantitatively measured the accuracies of the human detection based on precision and recall as shown in Equations (11) and (12) [8,36].

(11) $$\text{Precision}=\;\frac{\#\text{TP}}{\#\text{TP}+\#\text{FP}}$$

(12) $$\text{Recall}=\;\frac{\#\text{TP}}{\#\text{human regions in all the images}}$$

where #TP, #FP and #human regions in all the images show the number of TP cases, FP cases, and human regions in all the images, respectively. As shown in Equations (11) and (12), the maximum and minimum values of both precision and recall are 1 and 0, respectively. The higher values (closed to 1) represent a higher accuracy of human detection. In Table 4, we can see that the accuracies of human detection in our system are high for various environments. However, the recall at night is comparatively lower than that of the other cases because no information from visible light image can be obtained, as shown in the left image of Figure 12c. 

**Table 4:** Detection results using dual camera systems.

Environment	#Frame	#People	#TP	#FP	Recall (%)	Precision (%)
Morning	500	899	786	15	87.43	98.13
Afternoon	500	730	677	5	92.74	99.27
Night	500	698	561	27	80.37	95.41
Rainy day	500	559	544	2	97.32	99.63
Total	2000	2886	2568	49	88.98	98.13

In addition, we compared the accuracies of our system of dual cameras with those of only visible light or thermal cameras; the results are shown in Table 4, Table 5 and Table 6. As shown in Table 4, Table 5 and Table 6, the accuracies of our system are much higher than those of only visible light or thermal cameras for all the cases, namely, mornings, afternoons, nights, and rainy days.

**Figure 13:**
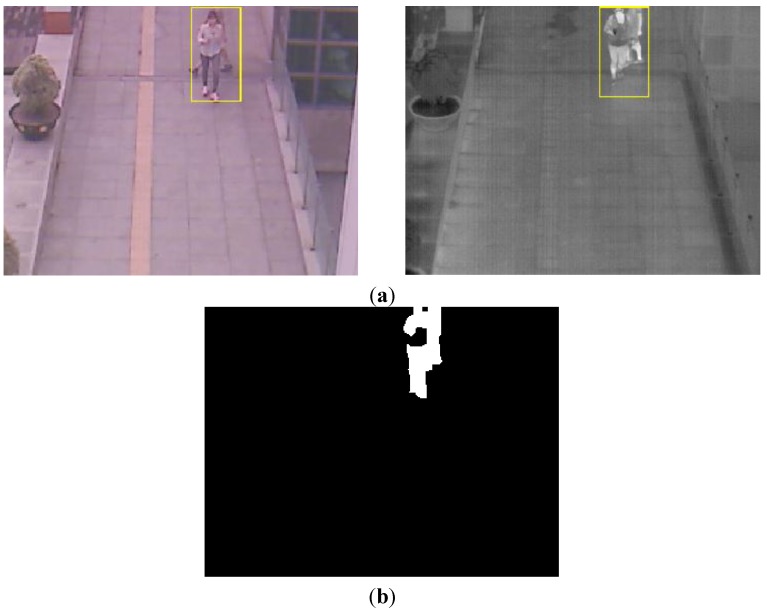
Detection error case in our database: (**a**) The example of the current input images by visible light (left figure) and thermal cameras (right figure); (**b**) Result image (of Step (17) of Figure 3).

In Figure 13, we show the detection error case by the proposed method. As shown in Figure 13, the error cases happen when occlusion by two pedestrians exists, which would be solved by using tracking information as future work.

**Table 5:** Detection result using only visible light camera.

Environment	#Frame	#People	#TP	#FP	Recall (%)	Precision (%)
Morning	500	899	556	11	61.85	98.06
Afternoon	500	730	594	9	81.37	98.51
Night	500	698	0	0	0	Cannot be calculated
Rainy day	500	559	254	523	45.44	32.69
Total	2000	2886	1404	543	48.65	72.11

**Table 6:** Detection result using only thermal camera.

Environment	#Frame	#People	#TP	#FP	Recall (%)	Precision (%)
Morning	500	899	759	22	84.43	97.18
Afternoon	500	730	252	2	34.52	99.21
Night	500	698	554	64	79.37	89.64
Rainy day	500	559	543	2	97.14	99.63
Total	2000	2886	2108	90	73.04	95.91

As the next experiment, we measured the processing time of our method as shown in Table 7. As shown in Table 7, the total processing time is about 23.13 ms and we find that our system can be operated at the speed of about 43.23 frames/s (1000/23.13).

**Table 7:** Processing time of our method.

Steps of Figure 3	Processing Time (ms)
Steps (1)–(4), (7) and (8)	16.05
Steps (5) and (6)	2.44
Steps (9)–(12)	2.25
Step (13)	0.25
Steps (14) and (15)	0.72
Steps (16)–(18)	1.42
Total	23.13

As the next test, we compare our algorithm with other already published methods [8,14,22]. Although HOG detector [8,14] and other detector using shape-based and appearance-based features [22] have been used in previous researches, the former method [8,14] has the disadvantage that it takes processing time for extracting the gradient information of various directions. In addition, the additional classifier based on SVM should be used with the HOG features, which requires additional (time-consuming) procedure of training [8,14]. The latter method [22] has the disadvantage that it takes processing time for extracting the skeleton information as the shape-based feature. In addition, the additional (time-consuming) procedure of training for principal component analysis (PCA) is required for extracting the appearance-based feature [22]. In all these methods, the training procedures of SVM and PCA makes the performance of system affected by the training data, also.

Because their method is for the pedestrian detection and tracking in thermal image [22], we compared the performance by our method in thermal image (Table 6) and that by their method. As shown in Table 6 and Table 8, average recall and precision by our method are higher than those by previous method [22]. In addition, we compared the processing time of our method with that by previous method [22]. The total processing time of our method is 23.13 ms (Table 7) which is smaller than that by previous method (48.54 ms). From these results, we can confirm that our method outperforms the previous one [22].

**Table 8:** Detection result using only thermal camera by previous method [22].

Environment	#Frame	#People	#TP	#FP	Recall (%)	Precision (%)
Morning	500	899	785	57	87.32	93.23
Afternoon	500	730	247	27	33.84	90.15
Night	500	698	517	131	74.07	79.78
Rainy day	500	559	541	37	96.78	93.60
Total	2000	2886	2090	252	72.42	89.24

In the next experiment, we compared the performance by our method with that by previous method [8,14]. Because their method can be applied to both the visible light and thermal images [8,14], we compared the performances by our method in visible light (Table 5) and thermal image (Table 6) and those by their method. As shown in Table 5 and Table 9, average recall and precision in visible light image by our method are higher than those by previous method [8,14]. In addition, as shown in Table 6 and Table 10, average recall and precision in thermal image by our method are higher than those by previous method [8,14]. In addition, we compared the processing time of our method with that by previous method [8,14]. The total processing time of our method is 23.13 ms (Table 7) which is smaller than that by previous method (42.57 ms). From these results, we can confirm that our method outperforms the previous one [8,14].

**Table 9:** Detection result using only visible light camera by previous method [8,14].

Environment	#Frame	#People	#TP	#FP	Recall (%)	Precision (%)
Morning	500	899	579	33	64.40	94.61
Afternoon	500	730	560	46	76.71	92.41
Night	500	698	0	0	0	Cannot be calculated
Rainy day	500	559	248	501	44.36	33.11
Total	2000	2886	1387	580	48.06	70.51

**Table 10:** Detection result using only thermal camera by previous method [8,14].

Environment	#Frame	#People	#TP	#FP	Recall (%)	Precision (%)
Morning	500	899	626	7	69.63	98.89
Afternoon	500	730	242	61	33.15	79.87
Night	500	698	507	10	72.64	98.07
Rainy day	500	559	429	2	76.74	99.54
Total	2000	2886	1804	80	62.51	95.75

**Figure 14:**
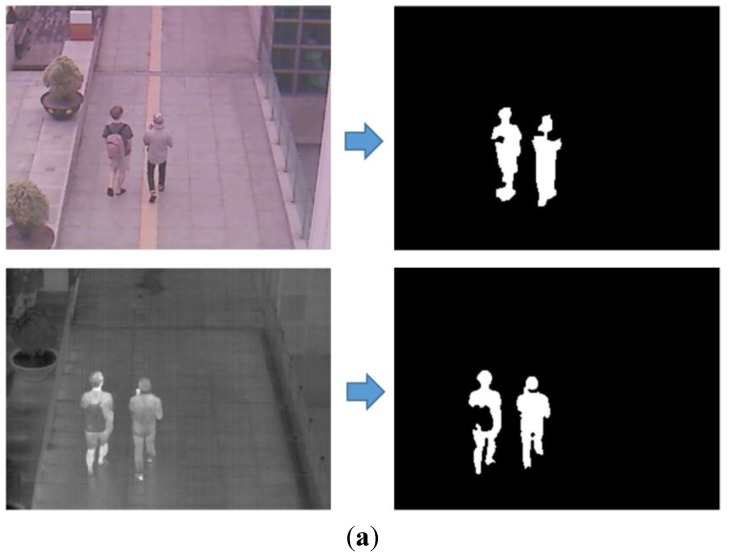

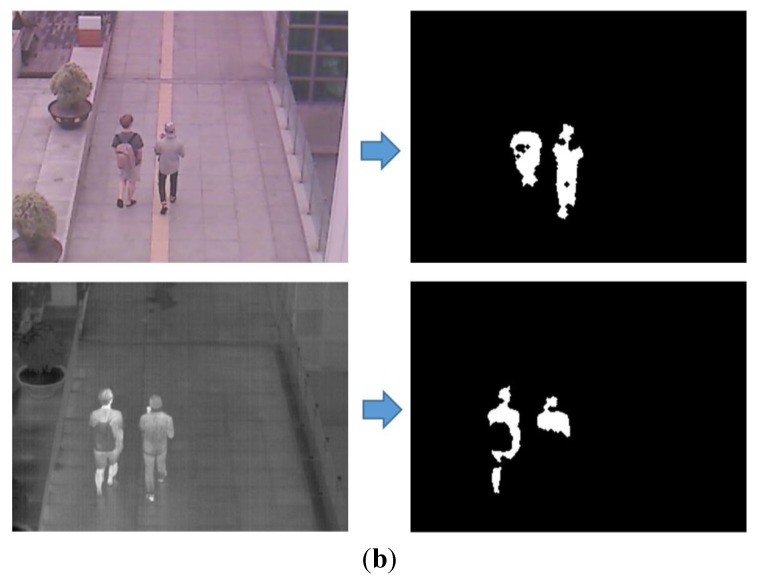
The results of background subtraction by our method and previous one [37]. Upper and lower figures of (**a**,**b**) are the results with the visible light and thermal images, respectively: (**a**) Results by our method; (**b**) Results by previous method [37].

In addition, we compared the background subtraction by our method with that based on Gaussian background-subtraction approach which has been widely used [37]. For fair comparisons, only the background update and subtraction (Steps (1)–(5) and (7)–(9) of Figure 3) are replaced by [37] when measuring the performance by previous method [37]. Because their method can be applied to both the visible light and thermal images, we compared the performances by our method in visible light (Table 5) and thermal image (Table 6) and those by their method. Figure 14 shows the results of background subtraction by our method and previous one [37]. As shown in this figure, we can find that our background subtraction method outperforms the previous one [37].

As shown in Table 5 and Table 11, average recall and precision in visible light image by our background subtraction method are higher than those by previous method [37]. In addition, as shown in Table 6 and Table 12, average recall and precision in thermal image by our background subtraction method are higher than those by previous method [37]. In addition, we compared the processing time of our background subtraction method with that by previous method [37]. The total processing time of our method is 16.84 ms (Steps (1)–(5), and (7)–(9) of Table 7) which is smaller than that by previous method (26.27 ms) [37]. From these results, we can confirm that our background subtraction method outperforms the previous one [37].

**Table 11:** Detection result using only visible light camera by previous method [37].

Environment	#Frame	#People	#TP	#FP	Recall (%)	Precision (%)
Morning	500	899	464	32	51.61	93.55
Afternoon	500	730	573	17	78.49	97.12
Night	500	698	0	0	0	Cannot be calculated
Rainy day	500	559	143	445	25.58	24.32
Total	2000	2886	1180	494	40.89	70.49

**Table 12:** Detection result using only thermal camera by previous method [37].

Environment	#Frame	#People	#TP	#FP	Recall (%)	Precision (%)
Morning	500	899	500	379	55.62	56.88
Afternoon	500	730	406	109	55.62	78.84
Night	500	698	590	43	84.53	93.21
Rainy day	500	559	109	653	19.50	14.30
Total	2000	2886	1605	1184	55.61	57.55

**Figure 15:**
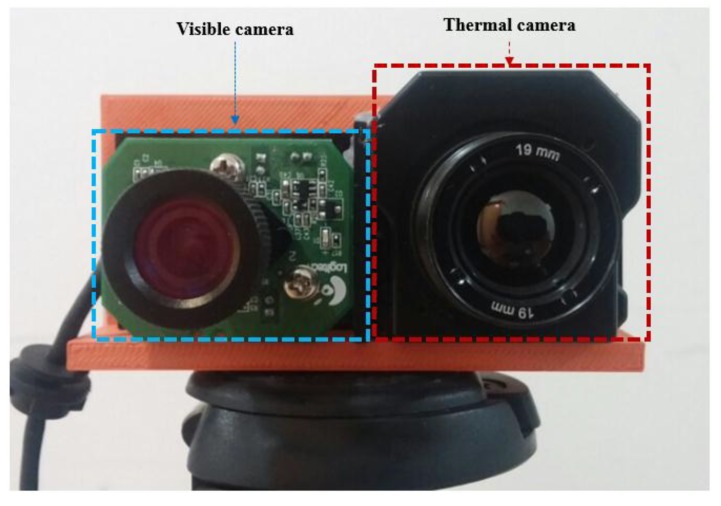
Proposed dual camera system which is used for collecting database II.

In order to prove that our method is robust to the kinds of camera and database, we measured the performance by our method with another database. This additional database (database II) is collected by a new dual camera system as shown in Figure 15. The total number of images used in the experiment (database II) was 800. These images were obtained in various environments such as mornings of 2.9 °C (200 images), afternoons of 4.7 °C (200 images), nights of 1.2 °C (200 images), and rainy days of 2.8 °C with precipitation of 5.5 mm (200 images). We captured the images where people naturally move without any instruction from us. Therefore, there exist various cases that some people are close together, cluttered, separated, *etc*. in our database.

Like the first system of dual cameras in Figure 1, we implement the dual camera system by combining visible light and thermal cameras in order to collect database II. A commercial thermal camera of FLIR Tau2 is used [38]. It can capture an image of 640 × 480 pixels having a resolution of 8 bits in the wavelength range of 7.5–13.5 μm. A 19 mm lens is used in the thermal camera, and the field of view (FOV) of the thermal camera is 32° and 26° in the horizontal and vertical directions, respectively. The dimension (height × width × depth) and weight of the thermal camera are 1.75" × 1.75" × 1.18" and approximately 70 g, respectively. 

**Figure 16:**
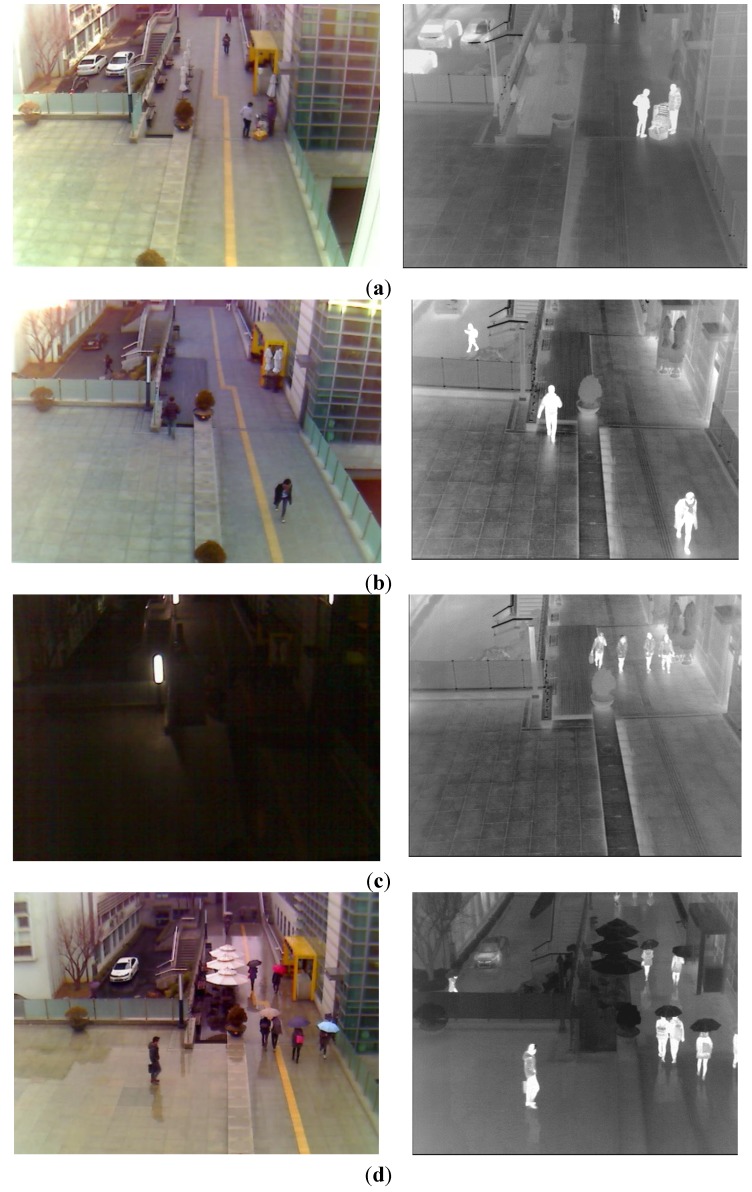
Examples of collected images in database II. Left and right figures of (**a**–**d**) are the images by visible light and thermal cameras, respectively. Image captured (**a**) in the morning; (**b**) in the afternoon; (**c**) at night; (**d**) on a rainy day.

The same web-camera of Figure 1 is used as the visible light camera [30]. The FOV of the visible light camera using a 6 mm lens is 42° and 32° in the horizontal and vertical directions, respectively, which is much wider than that of the visible light camera of Figure 1. Our system acquires both the visible light image of 800 × 600 pixels and the thermal image of 640 × 480 pixel at the capturing speed of 30 frames per sec. By using the lenses of wider FOV for the visible light and thermal cameras of Figure 15 than those of Figure 1, our additional database (database II) includes the images of wider FOV compared to those by the system of Figure 1 as shown in Figure 12 (database I) and Figure 16 (database II). However, the size of people in the database II (Figure 16) becomes smaller than that in database I (Figure 12) due to the wider FOV.

In order to reduce the image disparity between the two cameras, we make the two axes of visible light and thermal cameras parallel in the horizontal direction with minimum horizontal distance between the two cameras as shown in Figure 15.

In Table 13, Table 14 and Table 15, we show the accuracies by our method with database II.

**Table 13:** Detection results using dual camera systems by our method with database II.

Environment	#Frame	#People	#TP	#FP	Recall (%)	Precision (%)
Morning	200	167	135	1	80.84	99.26
Afternoon	200	216	210	26	97.22	88.98
Night	200	269	254	2	94.42	99.22
Rainy day	200	181	180	72	99.45	71.43
Total	800	833	779	101	93.52	88.52

**Table 14:** Detection result using only visible light camera by our method with database II.

Environment	#Frame	#People	#TP	#FP	Recall (%)	Precision (%)
Morning	200	167	48	16	28.74	75.00
Afternoon	200	216	132	28	61.11	82.50
Night	200	269	0	0	0	Cannot be calculated
Rainy day	200	181	142	70	78.45	66.98
Total	800	833	322	114	38.66	73.85

**Table 15:** Detection result using only thermal camera by our method with database II.

Environment	#Frame	#People	#TP	#FP	Recall (%)	Precision (%)
Morning	200	167	128	55	76.65	69.95
Afternoon	200	216	149	119	68.98	55.60
Night	200	269	241	35	89.59	87.32
Rainy day	200	181	180	5	99.45	97.30
Total	800	833	698	214	83.79	76.54

For the next experiment, we measured the processing time of our method with database II as shown in Table 16. As shown in Table 16, the total processing time is about 27.04 ms and we find that our system can be operated at the speed of about 36.98 frames/s (1000/27.04). By comparing the processing time of Table 7, the processing time of Steps (1)–(4), (7) and (8) in Table 16 is much smaller than that of Table 7. That is because the thermal image in database I (used in Table 7) includes a lot of noises and additional median filtering is included in the Steps (1)–(4), (7) and (8) in Table 7.

As the next test, we compare our algorithm with previous detectors [8,14,22] with database II. Because their method is for the pedestrian detection and tracking in thermal image [22], we compared the performance by our method in thermal image (Table 15) and that by their method. As shown in Table 15 and Table 17, average recall and precision by our method are higher than those by previous method [22]. In addition, we compared the processing time of our method with that by previous method [22]. The total processing time of our method is 27.04 ms (Table 16) which is smaller than that by previous method (59.14 ms). From these results, we can confirm that our method outperforms the previous one [22].

**Table 16:** Processing time of our method with database II.

Steps of Figure 3	Processing Time (ms)
Steps (1)–(4), (7) and (8)	0.003
Steps (5) and (6)	1.60
Steps (9)–(12)	18.10
Step (13)	0.97
Steps (14) and (15)	5.69
Steps (16)–(18)	0.68
Total	27.04

**Table 17:** Detection result using only thermal camera by previous method [22] with database II.

Environment	#Frame	#People	#TP	#FP	Recall (%)	Precision (%)
Morning	200	167	80	103	47.90	43.72
Afternoon	200	216	177	83	81.94	68.08
Night	200	269	206	52	76.58	79.85
Rainy day	200	181	150	10	82.87	93.75
Total	800	833	613	248	73.59	71.20

In the next experiment, we compared the performance by our method with that by previous detector [8,14]. Because their method can be applied to both the visible light and thermal images [8,14], we compared the performances by our method in visible light (Table 14) and thermal image (Table 15) and those by their method. As shown in Table 14 and Table 18, average recall and precision in visible light image by our method are higher than those by previous method [8,14]. In addition, as shown in Table 15 and Table 19, average recall and precision in thermal image by our method are higher than those by previous method [8,14]. In addition, we compared the processing time of our method with that by previous method [8,14]. The total processing time of our method is 27.04 ms (Table 16) which is smaller than that by previous method (54.55 ms). From these results, we can confirm that our method outperforms the previous one [8,14].

**Table 18:** Detection result using only visible light camera by previous method [8,14] with database II.

Environment	#Frame	#People	#TP	#FP	Recall (%)	Precision (%)
Morning	200	167	32	20	19.16	61.54
Afternoon	200	216	117	45	54.17	72.22
Night	200	269	0	0	0	Cannot be calculated
Rainy day	200	181	147	92	81.22	61.51
Total	800	833	296	157	35.53	65.34

**Table 19:** Detection result using only thermal camera by previous method [8,14] with database II.

Environment	#Frame	#People	#TP	#FP	Recall (%)	Precision (%)
Morning	200	167	108	48	64.67	69.23
Afternoon	200	216	121	98	56.02	55.25
Night	200	269	237	44	88.10	84.34
Rainy day	200	181	177	19	97.79	90.31
Total	800	833	643	209	77.19	75.47

In addition, we compared the background subtraction by our method with that based on Gaussian background-subtraction approach which has been widely used [37] with database II. For fair comparisons, only the background update and subtraction (Steps (1)–(5) and (7)–(9) of Figure 3) are replaced by [37] when measuring the performance by previous method [37]. Because their method can be applied to both the visible light and thermal images, we compared the performances by our method in visible light (Table 14) and thermal image (Table 15) and those by their method. 

As shown in Table 14 and Table 20, average recall and precision in visible light image by our background subtraction method are higher than those by previous method [37]. In addition, as shown in Table 15 and Table 21, average recall and precision in thermal image by our background subtraction method are higher than those by previous method [37]. In addition, we compared the processing time of our background subtraction method with that by previous method [37]. The total processing time of our method is 7.73 ms (Steps (1)–(5), and (7)–(9) of Table 16) which is smaller than that by previous method (51.54 ms). From these results, we can confirm that our background subtraction method outperforms the previous one [37]. 

**Table 20:** Detection result using only visible light camera by previous method [37] with database II.

Environment	#Frame	#People	#TP	#FP	Recall (%)	Precision (%)
Morning	200	167	27	0	16.17	100
Afternoon	200	216	118	28	54.63	80.82
Night	200	269	0	0	0	Cannot be calculated
Rainy day	200	181	102	73	56.35	58.29
Total	800	833	247	101	29.65	70.98

**Table 21:** Detection result using only thermal camera by previous method [37] with database II.

Environment	#Frame	#People	#TP	#FP	Recall (%)	Precision (%)
Morning	200	167	129	50	77.25	72.07
Afternoon	200	216	139	124	64.35	52.85
Night	200	269	178	34	66.17	83.96
Rainy day	200	181	180	5	99.45	97.30
Total	800	833	626	213	75.15	74.61

In our system, the background image (where no human area is included) is manually saved, and this procedure is performed one time only at the initial setup of our system. If the human area exists in the current input image, the pixel difference between the input and background images becomes large (the condition of Step 2 of Figure 3 makes a result of “Yes”), and the background update is not performed as shown in the Step 2 of Figure 3, consequently. Therefore, even in the case that the human area remains steady in the current input images, the initial background (not including the human area) is not updated due to the condition of the Step 2 of Figure 3, and the human areas can be detected by our background subtraction between the input and background images.

In previous researches [39,40], Serrano-Cuerda *et al.*, proposed the method of human detection by the fusion of visible light and thermal videos. In their method, the detection result from the thermal or visible light images is adaptively selected based on the confidence level of the thermal or visible light image. Castillo *et al.*, proposed the method of detecting dangerous situations at home, such as falls, by using color and thermal cameras [41]. In their method, the behavior of fall is recognized based on fuzzy system, and the recognition is done independently on the indoor images of visible light and thermal cameras, respectively. The two decisions (Yes or No) of the behavior of fall from the images of visible light and thermal cameras are combined in decision level fusion.

In another research [42], they proposed the multi-agent system for infrared and color video fusion. They showed the method of adaptively selecting the detection result from the thermal or visible light images based on the confidence level of the thermal or visible light image like the researches [39,40]. However, they did not show the detail method of refining the detected area of human by fusing the positional information of two detected areas of human in visible light and thermal images. In other research [43], they proposed the method of extracting the ROI of human by combining the information of binarized input image and motion. They used only the thermal image without the visible light ones. 

The main characteristics of these researches are that they adaptively select the detection result of human from the thermal or visible light images based on the confidence level of the thermal or visible light image. The confidence level of the image is determined by the average gray value of the input image by visible light camera and the ratio of the average gray value of the input image by thermal camera to the standard deviation of the input image by thermal camera. However, in our research, as shown in Figure 7 and Figure 9, we fuse the two detected areas (CWI and CSI) of human in visible light and thermal images by the mapping of pixel positions based on the geometric transform (of Equations (1) and (2)) between two cameras as shown in Figure 3. In addition, we quantitatively measured the accuracy of mapping of pixel positions based on the geometric transform as shown in Figure 10 and Figure 11 and Table 2 and Table 3. By combining the two detected areas of human as shown in the Step (16) of Figure 3, more refined areas of human can be obtained by our method. This is the 1st and main difference between our method and previous researches. 

For the second difference between our method and these previous researches, we adaptively update two background images for visible light and thermal cameras when the pixel difference between an input thermal image and pre-stored thermal background image is smaller than the threshold whereas the previous method used adaptive Gaussian background modeling [40]. In addition, we do not use the scheme of frame subtraction (motion information) whereas the previous researches adopted this scheme [39,40,43]. These schemes of Gaussian background modeling and frame subtraction have the disadvantage that they cannot cover the cases that human is not moved in all the frames. We overcome this disadvantage by fusing the two detected areas (CWI and CSI) of human in visible light and thermal images, and these two areas are obtained by adaptive updating of the background image (Steps (4) and (8) of Figure 3) and background subtraction (Steps (5), (9) and (14) of Figure 3). 

In Figure 14 and Table 5, Table 6, Table 11 and Table 12, we compared the accuracies of human detection with our method and Gaussian background modeling [37]. As shown in Figure 14 and Table 11 and Table 12, we find that the accuracy in our method is higher than that by Gaussian background modeling. As mentioned before, the total processing time of our method is 7.73 ms (Steps (1)–(5), and (7)–(9) of Table 7) which is smaller than that by previous method (51.54 ms) [37]. From these results, we can confirm that our background subtraction method outperforms the previous one [37].

For the third difference, by using the background subtraction based on the adaptive thresholding for binarization considering the background temperature as shown in Equations (4)–(7), we can obtain the candidate region of human, which is less affected by the temperature of background. 

For the last difference between our method and these previous researches, in addition to the accuracies of human detection, we provide the processing speed of our method as shown in Table 7 considering the real-time application of intelligent surveillance system. However, previous researches did not show the results of processing speed [39,40,41,42,43].

The final goal of our research is to recognize the behavior of people in various environments outdoors, and this will be our next research. However, in the OSU thermal pedestrian database, OSU color-thermal database, and terravic motion IR database of OTCBVS dataset collection [33], the people size is so small that they are difficult to be used for behavioral recognition. In addition, in the pedestrian infrared/visible stereo video dataset of OTCBVS dataset collection [33], although the people size is large enough for behavioral recognition, they are collected indoors (not in various environments of outdoors). Therefore, we used our own database (database I) of 2000 images collected in various outdoor environments (morning, afternoon, nights and rainy day) as shown in Figure 12. In addition, we collected the additional database (database II which includes 800 images) as shown in Figure 16 for the experiments by using the dual cameras of wide FOV. Through the experimental results of Table 4, Table 5, Table 6, Table 7, Table 8, Table 9, Table 10, Table 11, Table 12, Table 13, Table 14, Table 15, Table 16, Table 17, Table 18, Table 19, Table 20 and Table 21 and Figure 12, Figure 13, Figure 14, Figure 15 and Figure 16 with databases I and II, we can confirm our method is robust to the kinds of camera and database, and the performance of our method is better than previous ones [8,14,22,37].

## 4. Conclusions

In this research, we proposed a method for detecting pedestrians using a dual camera system by combining visible light and thermal cameras. We implemented the dual camera system where the two camera axes were horizontally parallel, and obtained the geometric transform matrix that represents the relationship between these two camera axes. Two background images for visible light and thermal cameras were adaptively updated when the pixel difference between an input thermal image and pre-stored thermal background image was smaller than the threshold. By background subtraction and further processing of visible light and thermal images, the CWI and CSI of pedestrians were located in these two images. Then, the final areas of pedestrian were detected by combining the CWI and CSI based on the OR operation. Through experiments in various environments, we proved the effectiveness of our system. 

In future work, we would apply our results of human detection to the field of behavioral recognition. In addition, we would research a method of combining the information from multiple dual camera systems.
